# Assessment of the Access AMH assay as an automated, high-performance replacement for the AMH Generation II manual ELISA

**DOI:** 10.1186/s12958-016-0143-3

**Published:** 2016-02-16

**Authors:** Kylie Pearson, Matthew Long, Josephine Prasad, Ye Ying Wu, Michael Bonifacio

**Affiliations:** Genea-Canberra, 17B/2 King Street, Deakin, ACT 2600 Australia; Genea-Sydney City, Level 3/321 Kent Street, Sydney, NSW 2000 Australia; Genea-South West, 173-175 Bigge Street, Liverpool, NSW 2170 Australia

**Keywords:** Anti-Müllerian Hormone, AMH, Assay, Immunoassay, ELISA, Beckman Coulter, Automated, Reference range

## Abstract

**Background:**

The manual Generation II (Gen II) ELISA method used to measure Anti-Müllerian Hormone (AMH) from Beckman Coulter has recently been superseded by a fully automated AMH immunoassay. The aim of this study was to evaluate the performance of the Access AMH assay and directly compare it to the modified Gen II ELISA method. A secondary aim was to verify that the fertile age-related AMH range previously established using the Gen II ELISA could be used to interpret results from the new automated Access assay.

**Methods:**

The precision, stability, linearity, measurement range and detection limits were determined using recombinant AMH and patient serum samples. Different diluents and their effects on AMH concentration were compared. A correlation study was performed on patient samples to compare the Access AMH assay to the ELISA method on the Access2 and DxI800 analysers. The fertile AMH range was verified by comparing the 10th, 50th and 90th percentile values from both methods obtained from 489 natural conception pregnant women.

**Results:**

The Access AMH assay showed good performance across the measuring range for both intra-assay (CV 1.41–3.30 %) and inter-assay (CV 3.04–5.76 %) precision and acceptable sample stability. Dilution of the high concentration samples with the recommended diluent resulted in a small but significant downward shift in values. The assay was linear over the range of values recommended by the manufacturer, allowing for accurate reporting within the reported range. The two assay types were highly correlated (R^2^ = 0.9822 and 0.9832 for Access2 and DxI800, respectively), and the differences observed between the Access2 and DxI800 analysers were within clinically acceptable ranges, indicating that the methods are interchangeable. Furthermore, we demonstrated that results from the published reference range for the Gen II ELISA correlate with those from the automated Access AMH assay.

**Conclusion:**

Here, we verified the published performance of the Access AMH assay and showed excellent correlation with the Gen II ELISA method. Moreover, we validated this correlation by confirming that the results from a fertile AMH reference range established using the preceding Gen II ELISA are interchangeable with the new automated Access AMH assay.

**Electronic supplementary material:**

The online version of this article (doi:10.1186/s12958-016-0143-3) contains supplementary material, which is available to authorized users.

## Background

Anti-Müllerian hormone (AMH) is expressed by the ovarian granulosa cells of the female ovary where it has important autocrine and paracrine regulator functions in follicle development. It is predominantly produced by the pre-antral and small antral follicles, and production then declines during the final maturation process and luteal phase [1]. AMH acts as an inhibitor of further follicle recruitment and inhibits the response of larger follicles to follicle-stimulating hormone (FSH)-induced growth and selection [2, 3].

The number of growing follicles and the resultant level of AMH released into the circulation is proportionate to the size of the primordial follicle stock [4, 5]. While other tests for ovarian reserve such as basal FSH remain valuable to fertility investigation particularly for patients with reduced ovarian reserve [6, 7], the serum concentration of AMH is gonadotropin independent, thus it remains relatively constant throughout the menstrual cycle [8, 9]. Consequently, AMH serum concentration has emerged as a unique biological marker for the size of the residual follicular pool that exhibits high correlation with ovarian reserve [10]. This single blood test can additionally assist in the prediction of ovarian response to stimulation, aid in the diagnosis of polycystic ovary syndrome (PCOS) and predict premature ovarian failure, among other uses [11–14].

A number of AMH immunoassays have been developed in the past; however, a lack of standardisation and technical issues between different methods has led to confusion in the interpretation of results and scepticism of AMH test reliability [15–17]. We recently established an AMH reference range of fertile women using the widely-used modified AMH Gen II enzyme-linked immunosorbent assay (ELISA) from Beckman Coulter [18]. The antibodies used in the Gen II assay have now been adopted in the first commercial fully automated AMH assay systems from Roche Diagnostics (Elecsys) and Beckman Coulter (Access) for both the Access2 and DxI800 instruments [19, 20]. Studies have revealed good correlations between the Gen II and Elecsys assays; however, a consensus on correlation between the Gen II ELISA and the new Access AMH assay has not been reached [21, 22].

In the present study, we assessed the accuracy and reproducibility of the Access AMH assay from Beckman Coulter and performed a correlation study with the previous Gen II ELISA method. Furthermore, we determined whether our published age reference range of AMH values from fertile women could be used to interpret results from the new AMH assay.

## Methods

The Beckman Coulter Access AMH immunoassay was assessed for use on both the Beckman Coulter Access2 and DxI800 analysers. Assay precision was evaluated for both intra- and inter-run precision using AMH quality control (QC) material consisting of human recombinant AMH (Beckman Coulter) at three known concentrations. Aliquots of samples were frozen and thawed once prior to testing. Intra-assay performance of the Access AMH assay was determined from 10 replicates of the assay QC material during the same running cycle, and inter-assay performance was determined by analysing the first run of QC material each day for nine consecutive days. The data was calculated as CV % (standard deviation/mean × 100).

Sample stability was assessed on patient sera and QC material. For patient sera, blood was allowed to clot in SST tubes (Becton Dickinson), and the serum was separated by centrifugation according to the manufacturer’s recommendations before being stored at -20 °C until further analysis. Stability at 4 °C was determined by testing a sample from one patient in triplicate for eight consecutive days. Stability over freeze/thaw cycles was assessed by testing a fresh sample on day one, then freezing the sample at -20 °C and thawing prior to testing for seven cycles. The data was calculated as % deviation of mean (mean – expected mean/expected mean × 100).

Assay linearity was confirmed across the measuring range by testing dilutions of calibrator material (recombinant human AMH; Beckman Coulter) and patient sera. The S5 AMH calibrator was diluted out in a series using Sample Diluent A. The patient sera dilution series was performed using a mix of sera from patients with known high and low (<0.3 pmol/L) AMH concentrations to ensure that no matrix effect was present. Linearity was determined using the Cusum test for linearity.

Assay detection limits were determined using doubling dilutions of a patient serum sample with wash buffer (Beckman Coulter). The Limit of Blank (LoB) was calculated as the mean of the blank plus 1.645 times the standard deviation of the blank, while the Limit of Detection (LoD) was calculated as the LoB plus 1.645 times the standard deviation of low-level samples using the highest standard deviation value of the low-level samples tested.

Dilutions of three patient sera samples (AMH concentrations 75, 115 and 140 pmol/L; samples 1, 2 and 3 respectively) were tested in duplicate, comparing neat values with 1:5 dilutions in low concentration patient sera, the recommended diluent from Beckman Coulter (Sample Diluent A), or wash buffer. Dilution testing was also performed on patient sera of mid- and high-range concentrations (77 and 177 pmol/L), comparing neat values and samples diluted 1:5, 1:10 and 1:16 in Sample Diluent A or patient sera of AMH concentration <0.1 pmol/L. A set of patient samples of AMH concentration >70 pmol/L (*n* = 27) were assayed neat or diluted 1:5 in Sample Diluent A. The results were analysed by a two tailed *t-*test.

The correlation between the new automated Access AMH assay and the previous Beckman Coulter AMH Gen II ELISA: revised protocol [23] was determined on both the Access2 and DxI800 analysers. Sample AMH concentrations were determined by single measurements from 142 fresh patient serum samples by the Gen II ELISA. Then, the samples were stored at -20 °C and thawed once before measurement using the automated Access AMH method for each analyser. A further correlation of 46 samples stored at -20 °C was conducted between two separate Access2 analysers. Regression analysis was performed using Passing-Bablok and Bland-Altman methods of comparison [24, 25].

The fertile AMH reference range was previously determined [18]. Briefly, a prospective observational study was conducted on 492 pregnant women in their first trimester, aged between 20 and 44 years, who had all conceived spontaneously without the use of ovarian stimulation drugs within 2–3 months of attempted conception. Blood samples were taken and stored at -80 °C, and their AMH concentration was determined using the revised Gen II assay. To test for correlations between the reference ranges determined using the ELISA with the automated AMH assay, 489 of the original samples stored at -80 °C were thawed and tested on the DxI800 analyser using the automated AMH assay. The results were analysed to create an age-stratified collection of patient reference ranges. Patients were broken down into 5-year age brackets, and the 10th, 50th and 90th percentile AMH values of each bracket were calculated. These values were plotted using the median age of the patients for each bracket to create a polynomial curve for the limits of the AMH reference range.

Statistical calculations were carried out using Microsoft Excel 14.0 and SPSS Statistics 22.0 for the *t-*tests. Comparison studies were performed using Medcalc Version 15.11.4. All research was conducted in accordance with the ethics guidelines approved by the Genea Ethics Committee (EC00289) under approval GEC0028 with informed consent obtained from all participants.

## Results

### Precision

The Access assay showed good performance across the measuring range for the Access AMH Assay on both the Access2 and DxI800 instruments (Table 1). Intra-assay and inter-assay precision ranged from CV 1.41- 3.30 % and CV 3.04-5.76 %, respectively, and are within the ranges reported by Beckman Coulter.

**Table 1:** AMH intra- and inter-assay precision on Access2 and DxI800 analysers

		Intra-assay precision			Inter-assay precision		
		Mean, pmol/L	SD, pmol/L	CV, %	Mean, pmol/L	SD, pmol/L	CV, %
Access2	Control 1	6.27	0.09	1.51	6.23	0.24	3.85
	Control 2	31.78	0.45	1.41	31.69	0.96	3.04
	Control 3	97.85	1.64	1.68	97.19	3.35	3.45
DxI800	Control 1	6.40	0.21	3.30	6.31	0.28	4.36
	Control 2	33.36	0.68	2.04	32.49	1.87	5.76
	Control 3	103.18	2.98	2.89	98.54	4.52	4.59

### Sample stability

Human recombinant QC material and patient sera stored at 4 °C were shown to be stable over this time period (deviation from day 1 mean: -0.67–3.58 %). The effect of up to five freeze/thaw cycles on sample stability was also minimal (deviation from day 1 mean: -0.11–3.80 %; Table 2).

**Table 2:** AMH stability under different storage conditions

	Stored at 4 °C				Freeze/thaw		
	Mean, pmol/L	SD, pmol/L	Deviation, %		Mean, pmol/L	SD, pmol/L	Deviation, %
Day 1	5.96	0.16	0.00	Fresh	5.96	0.16	0.00
Day 2	5.83	0.09	−2.29	x1	5.96	0.08	−0.11
Day 3	5.77	0.03	−3.24	x2	5.87	0.04	−1.57
Day 4	5.92	0.04	−0.67	x3	5.91	0.14	−0.89
Day 5	5.75	0.18	−3.58	x4	5.74	0.09	−3.80
Day 6	6.09	0.05	2.07	x5	5.81	0.18	−2.63
Day 7	5.75	0.01	−3.52	x6	5.74	0.04	−3.75
Day 8	5.83	0.22	−2.24	x7	5.78	0.07	−3.13

### Linearity

**Fig. 1:**
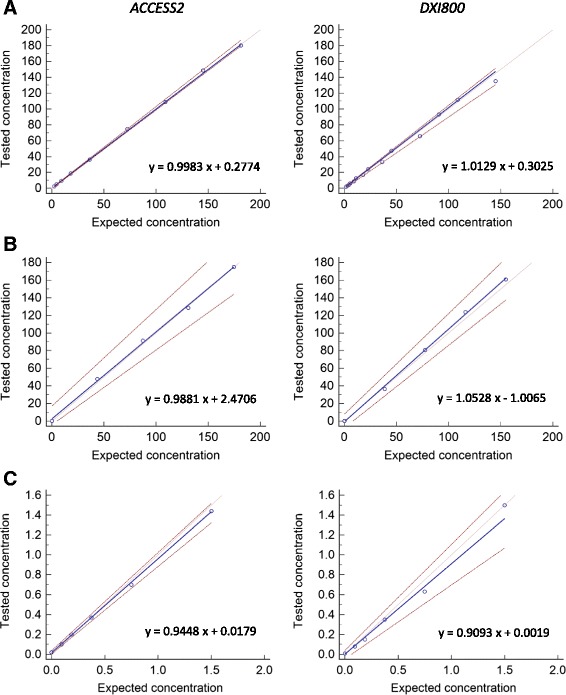
Linearity of the Access AMH assay on the Access2 and DxI800 analysers using **a**. calibrator material or **b**. patient sera. **c**. Linearity at the low end of the concentration range using patient sera. Regression line (blue), 95 % confidence intervals (red). All results are shown in pmol/L

The automated Access assay was established to be remarkably linear over the range of measurement values specified by Beckman Coulter when using either human recombinant AMH material or patient sera (Fig. 1a and b). Linear regression analysis yielded R^2^ values between 0.9947 and 0.9996 and slope values between 0.9881 and 1.0528. Furthermore, there was no significant deviation from linearity using the Cusum test for linearity under all tested conditions. Similar results were obtained using a dilution series of patient sera diluted in Sample Diluent A (unpublished observations). These data indicate that the linear relationship is maintained under the required assay conditions and thus allows for accurate reporting within the reported range.

### Assay limits

A dilution series of samples at the lower end of the measuring range resulted in good linearity on both the Access2 (R^2^ = 0.9995, slope = 0.9488) and DxI800 analysers (R^2^ = 0.9932, slope = 0.9093) (Fig. 1c). The LoD was calculated to be 0.1055 on the Access2 and 0.0994 on the DxI800, while the LoB was calculated to be 0.0200 on the Access2 and 0.0352 on the DxI800. All determined assay limits were lower than the values designated by Beckman Coulter (LoD = 0.14 pmol/L, LoB = 0.07 pmol/L) and confirmed the high sensitivity of this new assay.

### Dilution testing

A comparison of neat AMH concentrations with those obtained using three different dilution materials revealed that samples diluted in patient sera of low concentration showed the least deviation from the neat value, followed by Sample Diluent A and wash buffer (Additional file 1: Figure S1a). The dilution effect was further investigated by performing 1:5, 1:10 and 1:16 dilutions (1:16 being the recommended dilution factor from Beckman Coulter) on samples from patients with mid-range and high-range AMH concentrations and measuring AMH using the automated Access assay (Table 3). Dilution in low AMH concentration sera resulted in -2.56–5.21 % deviation from the mean neat result, while dilution in Sample Diluent A resulted in a deviation from the neat value of -3.63–13.31 %. This finding was confirmed by testing a cohort of patient samples of AMH concentration >70 pmol/L and comparing the neat results with the diluted (1:5 in Sample Diluent A) results (Additional file 1: Figure S1b). Overall, the results display a consistent decrease of approximately 10 % upon sample dilution, which, while significant (*t*-test *p* < 0.0001), would not be clinically relevant. These data suggest that there is no need for dilution of samples that are >70 pmol/L, as required for the previous Gen II ELISA; in fact, the opposite effect occurs upon dilution, where sample concentrations are negatively biased.

**Table 3:** Comparison of different dilution methods using the Access AMH Assay

		Dilution in patient sera			Dilution in sample diluent A		
	Dilution	Mean, pmol/L	CV, %	Deviation, %	Mean, pmol/L	CV, %	Deviation, %
Sample 1	Neat	76.90	0.50	0.00	76.90	0.50	0.00
	1:5	74.82	1.09	−2.71	68.90	1.73	−10.41
	1:10	74.63	1.59	−2.96	66.28	2.74	−13.81
	1:16	77.81	1.11	1.18	68.04	1.36	−11.52
Sample 2	Neat	170.64	1.83	0.00	170.64	1.83	0.00
	1:5	177.69	1.10	4.13	164.44	1.24	−3.63
	1:10	176.71	0.64	3.56	154.80	0.46	−9.28
	1:16	179.53	3.55	5.21	153.65	3.07	−9.96

### Correlation studies between assays and analysers

**Fig. 2:**
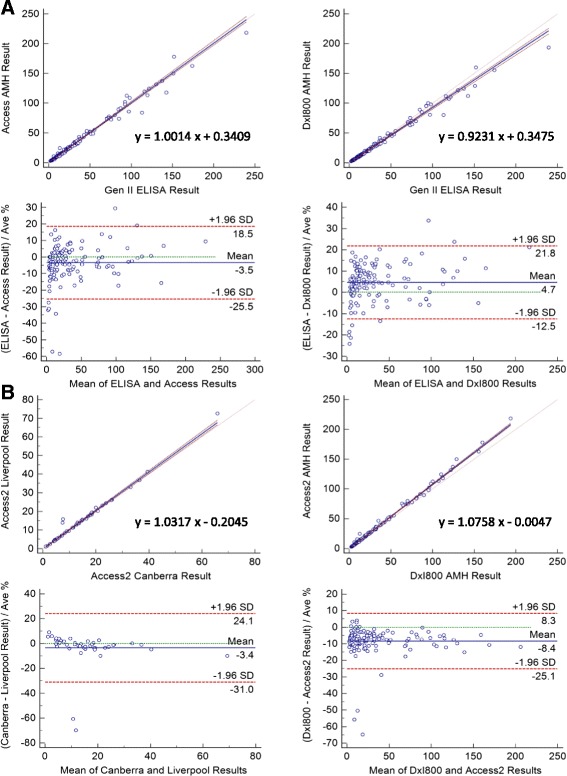
**a**. Correlation between Access AMH and Gen II ELISAs on the Access2 and DxI800 instruments (*n* = 142). **b**. Correlation between results from Access2 analysers in different locations (*n* = 46) and Access2 and DxI800 analysers (*n* = 142). Upper panels represent the Passing-Bablok diagram with the regression line (blue) and the 95 % confidence interval (red). Lower panels represent the Bland-Altman plots with mean (blue) and 95 % confidence intervals (red). All results are shown in pmol/L

The correlation of the new Access AMH assay with the preceding revised AMH Gen II ELISA was determined by a comparative study of 142 patient samples (Fig. 2a). Passing-Bablok regression analysis comparing the Gen II ELISA with the Access AMH assay run on the Access2 and DxI800 analysers resulted in R^2^ values of 0.9822 and 0.9832 and slope values of 1.0014 and 0.9231, respectively. All regression curves showed no significant deviation from linearity, indicating good correlation between these methods. The Bland-Altman analyses indicate an absence of bias across the analytical range for all comparisons.

The same patient cohort was used to perform a correlation study between the Access2 and DxI800 analysers using the Access AMH assay (R^2^ = 0.9964, slope = 1.0758). A smaller cohort of 46 patient samples was used to perform a comparison between two Access2 analysers located in different geographical locations (R^2^ = 0.9820, slope = 1.0317). Regression analysis and Bland-Altman plots demonstrate high correlation across the measuring range between the results obtained on the different instruments (Fig. 2b).

### Correlation study of normal AMH reference ranges

**Fig. 3:**
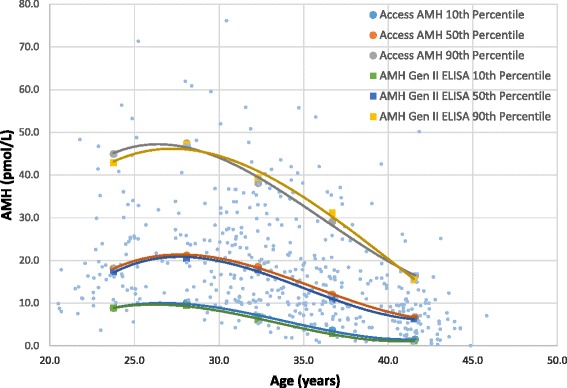
Correlation between automated AMH and Gen II ELISA normal female reference ranges. Individual results (blue points) and 10th, 50th and 90th percentile median values for 5-year age groups for Access AMH and Gen II ELISA methods (*n* = 489)

A large study cohort of 489 pregnant patients who were used previously to establish a fertile AMH reference range using the Gen II ELISA were re-tested using the Access AMH assay. The results from both assays were analysed to create an age-stratified collection of patient reference ranges by plotting the age group against the values of the median, 10th, 50th and 90th percentiles (Fig. 3). The results between the two methods were highly correlated (average CV 6.7 %), and the same significant curvilinear relationship between AMH and age was confirmed using the new AMH test. As a result, we conclude that the previously determined reference range can be used to interpret data from either assay.

## Discussion

AMH measurement is emerging as an extremely useful tool in a number of areas of reproductive medicine. However, technical issues with regards to stability and lack of standardisation between past AMH assays have led to uncertainty in AMH test reliability and result interpretation [15, 26, 27]. The findings of the correlation studies herein demonstrate agreement between the results generated by the AMH Gen II ELISA and the Access AMH Assay. Comparing the automated Access and manual ELISA method using the Access2 analyser showed near complete agreement, while results from the DxI800 analyser showed a slight difference within acceptable clinical ranges. These findings indicate that the past issues have been overcome when comparing the new and previous Beckman Coulter methods.

In support of our results, van Helden and Weiskirchen [22] demonstrated good correlations between the Gen II ELISA and both the Access AMH assay and the new automated Elecsys system from Roche, both of which utilise the same monoclonal antibodies. The authors also demonstrated an extremely tight correlation between the Access and Elecsys assays, and these results were further validated by Nelson et al. [21]. Interestingly, Nelson et al. [21] report a 22 % decrease in expected values and distinct systematic bias between the old and new Beckman Coulter AMH assays with increasing concentrations. Different sample storage and handling conditions may have contributed to this discordance. Hyldgaard et al. [28] demonstrated a similar pattern of bias between the Gen II and Elecsys methods and proposed that this may be in part due to inter-laboratory bias with the Gen II method. The Gen II ELISA is a manual method and thus would be more prone to inter-operator bias and variation from a number of sources within the assay itself. Our ELISAs were performed by a single experienced operator and we used a large sample size, which may account for the close correlation observed by our laboratory. Furthermore, we have shown that the dilution of high concentration samples and the use of different diluents can cause a shift in values, which may explain the conflicting results with high concentration samples.

Assessment of the Access AMH assay revealed excellent linearity and good performance across the measuring range for both intra-assay and inter-assay precision as would be expected for an automated immunoassay. This assay exhibited greatly increased sensitivity when compared to previous manual methods and aligned with literature from the manufacturer allowing for accurate reporting to 0.1 pmol/L. We demonstrated high levels of AMH immunoreactive stability under refrigerated and freeze/thaw conditions, though the long-term effect of storage under different conditions was not within the scope of this study. The results of the dilution testing revealed that AMH samples greater than 70 pmol/L do not need to be diluted as was required with the previous Gen II ELISA. In fact, our study revealed that dilution caused a negative shift of approximately 10 %, indicating care should be applied in the interpretation of results from diluted samples. This shift would not, however, have a significant effect on clinical outcome.

This is the first paper to report an established fertile age-related AMH reference range that is compatible with the automated Access AMH assay. Numerous studies have determined AMH reference ranges; however, the majority of this research was conducted using infertile or presumably fertile study groups and former methods [15, 16, 29–33]. Bonifacio et al. [18] recently published an AMH normogram using the revised Gen II ELISA method on a large cohort of first trimester pregnant patients who had conceived by natural and unaided means. AMH levels have been shown by a number of studies to exhibit little variation within and between menstrual cycles and to be stable from pre-pregnancy through the first trimester of pregnancy [34–36]; therefore, the study group was considered as representative of a fertile population. Here, we conducted a full study comprised of the same cohort using the automated Access AMH assay and the Gen II ELISA. A comparison of the results between the two assays showed variation within the performance limits of both analysis methods. This study further validated the results of our method comparison and confirmed that our fertile reference range of AMH values can be applied to both the previous ELISA and the new automated assay from Beckman Coulter.

## Conclusions

The two most important influences on the acquisition of reliable clinical information are the dependability of the measurements and the interpretation of the results. This study verified the published performance of the new automated Access AMH assay from Beckman Coulter showing a measuring range adequate for most IVF applications and the ability to detect values consistently across this range. The automated assay exhibited high levels of stability and sensitivity and showed correlation with the existing ELISA method and between analyser platforms. Furthermore, an age-related reference range of AMH values was established for patients not already undergoing infertility assessment, allowing for accurate extrapolation of data to tailor treatment and prognosis prediction in the wider population. These findings are an encouraging step towards the necessary establishment of universal clinically relevant cut-off values and the standardisation of AMH assay results.

## Additional file

Additional file 1: Figure S1. a. Effect of different diluents on the expected outcome of three patient samples. b. Effect of 1:5 dilution with Sample Diluent A on patient AMH concentration compared to neat values using the Access AMH assay. (PDF 25 kb)

## Abbreviations

AMH: Anti-Müllerian hormone
ELISA: Enzyme-linked immunosorbent assay
FSH: Follicle-stimulating hormone
Gen II: Generation II
IVF: In vitro fertilisation
LoB: limit of blank
LoD: limit of detection
PCOS: Polycystic ovary syndrome
QC: quality control material
