# Supplementary figures and images for: Assessment of the Access AMH assay as an automated, high-performance replacement for the AMH Generation II manual ELISA
Source: Reprod Biol Endocrinol. 2016 Feb 16;14:8. doi: 10.1186/s12958-016-0143-3 (PMC4754992; doi:10.1186/s12958-016-0143-3)

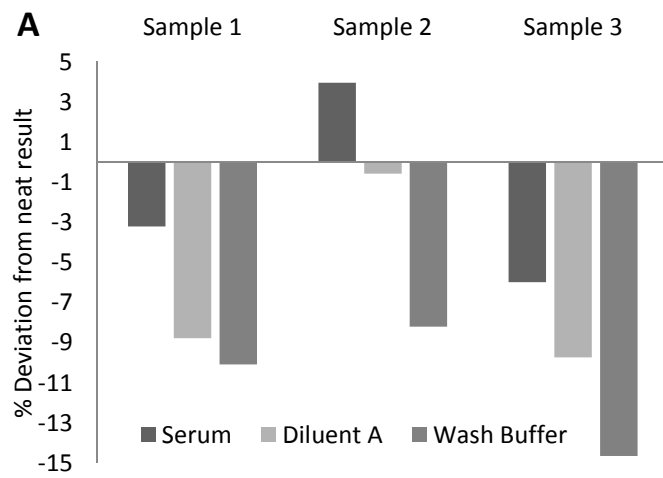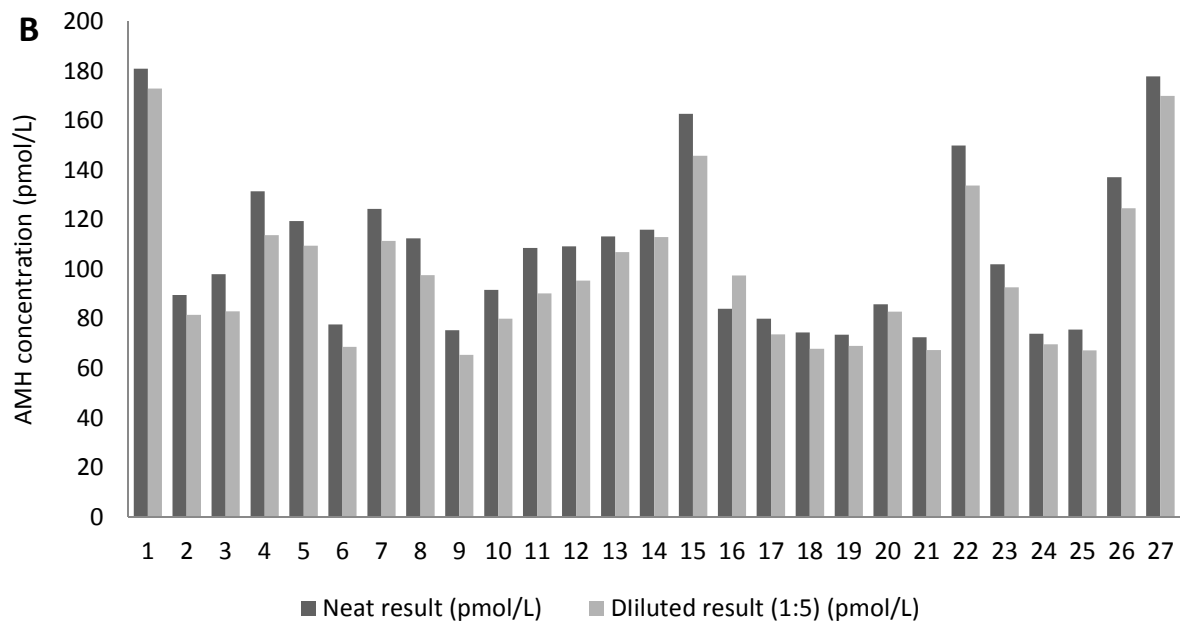

Supplement: Additional file 1: Figure S1. — a. Effect of different diluents on the expected outcome of three patient samples. b. Effect of 1:5 dilution with Sample Diluent A on patient AMH concentration compared to neat values using the Access AMH assay. (PDF 25 kb) [file 12958_2016_143_MOESM1_ESM.pdf]
